# Medicaid Enrollment and Service Use Among Adults With Down Syndrome

**DOI:** 10.1001/jamahealthforum.2023.2320

**Published:** 2023-08-11

**Authors:** Eric Rubenstein, Amy Michals, Na Wang, Ashley Scott, Salina Tewolde, A. Alex Levine, Yorghos Tripodis, Brian G. Skotko

**Affiliations:** 1 Department of Epidemiology, Boston University School of Public Health, Boston, Massachusetts; 2 Biostatistics and Epidemiology Data Analytics Center, Boston University School of Public Health, Boston, Massachusetts; 3 Department of Health Policy Law and Management, Boston University School of Public Health, Boston, Massachusetts; 4 Department of Biostatistics, Boston University School of Public Health, Boston, Massachusetts; 5 Down Syndrome Program, Massachusetts General Hospital, Boston, Massachusetts

## Abstract

**Importance:**

Down syndrome is the leading genetic cause of intellectual disability and automatically qualifies individuals for Social Security Insurance. Therefore, Medicaid is the major health insurance provider for a population at high risk for dementia, obesity, and premature mortality. Despite the importance of Medicaid for adults with Down syndrome, little is known about how this population uses Medicaid.

**Objective:**

To describe enrollment in, health care use in, and cost to Medicaid for adults with Down syndrome compared with adults with intellectual disability and a random sample of adults enrolled in Medicaid.

**Design, Setting, and Participants:**

In this cohort study, the data are from a claims cohort of adults aged 18 years or older enrolled in Medicaid at any point between January 1, 2011, and December 31, 2019. Participants were enrollees with 1 or more inpatient claim or 2 or more other claims with an *International Classification of Diseases, Ninth Revision* code or an *International Statistical Classification of Diseases and Related Health Problems, Tenth Revision *code for Down syndrome or intellectual disability as well as a random sample of those without developmental disability. Analyses were conducted from June 2022 to February 2023.

**Main Outcomes and Measures:**

Data were linked across 2 data reporting systems. Main outcomes were enrollee demographic characteristics, enrollment characteristics, cost, and service use.

**Results:**

This cohort study included 123 024 individuals with Down syndrome (820 273 person-years of coverage; mean [SD] age, 35 [14.7] years; median age, 33 years [IQR, 21-48 years]; 51.6% men; 14.1% Black individuals; 16.7% Hispanic individuals; and 74.6% White individuals), 1 182 246 individuals with intellectual disability (mean [SD] age, 37.1 [16.8] years; median age, 33 years [IQR, 22-50 years]; 56.5% men; 22.0% Black individuals; 11.7% Hispanic individuals; and 69.5% White individuals), and 3 176 371 individuals with no developmental disabilities (mean [SD] age, 38 [18.6] years; median age, 33 years [IQR, 21-52 years]; 43.8% men; 23.7% Black individuals; 20.7% Hispanic individuals; and 61.3% White individuals). Median enrollment in Medicaid for a person with Down syndrome was 8.0 years (IQR, 5.0-9.0 years; mean [SD], 6.6 [2.6] years). Costs were higher for the Down syndrome group (median, $26 278 per person-year [IQR, $11 145-$55 928 per person-year]) relative to the group with no developmental disabilities (median, $6173 per person-year [IQR, $868-$58 390 per person-year]). Asian, Black, Hispanic, Native American, and Pacific Islander adults with Down syndrome had fewer costs and claims per person-year compared with White adults with Down syndrome.

**Conclusion and Relevance:**

This cohort study of individuals with Down syndrome enrolled in Medicaid found consistent enrollment and high use of health care in a population with high health care needs. Results were similar comparing individuals with Down syndrome and those with intellectual disability, with both groups differing from a sample of Medicaid enrollees with no developmental disabilities. Medicaid data are a useful tool for understanding the health and well-being of individuals with Down syndrome.

## Key Points

**Question:**

What are the enrollment patterns and characteristics of adults with Down syndrome in the US Medicaid system, and how does that compare with other groups?

**Finding:**

In this cohort study from 2011 to 2019 of 123 024 adults with Down syndrome, 1 182 246 adults with intellectual disability, and a randomly selected comparison group of 3 176 371 individuals without developmental disabilities, adults with Down syndrome were more consistently enrolled in Medicaid and had higher costs and service use compared with the randomly selected comparison group.

**Meaning:**

This study suggests that the Medicaid program is vital for insuring adults with Down syndrome and facilitating access to health services for a community with high health care needs and costs.

## Teaser

This cohort study describes enrollment in, health care use in, and cost to Medicaid for adults with Down syndrome compared with adults with intellectual disability and a random sample of adults enrolled in Medicaid.

## Introduction

Down syndrome, defined by the partial or full trisomy of the 21st chromosome, is the leading genetic cause of intellectual disability. Down syndrome is present in approximately 1 in 800 live-born children,^1^ with an estimated 125 000 adults aged 18 years or older with Down syndrome in the US.^2^ With advances in treatment for heart defects, there has been a major increase in life expectancy for people with Down syndrome (median age of death, 1953: 4 years^2^; 2019: 57 years^3^). The longer life span for adults with Down syndrome is a major public health success but has implications for life-course health and the health care system because Down syndrome presents with co-occurring conditions.^4^ Nearly half of all adults with Down syndrome will develop Alzheimer disease or related dementias by 50 years of age, and all show signatures of dementia in postmortem autopsies.^5,6^

Medicaid is a major health insurer for adults with Down syndrome.^7^ With rare exception, all adults with Down syndrome are automatically eligible for Medicaid after enrolling in Social Security Insurance.^8^ Adults with Down syndrome could receive employer-based insurance coverage; however, systematic barriers to employment, such as disincentives and ableism, remain. Only approximately 3% of adults with Down syndrome are estimated to have full-time paid employment.^9^ Medicare, which can supplement Medicaid, is available for older adults and those who receive Social Security Disability Insurance (through their or a parent’s eligibility).^10^ Therefore, Medicaid provides a crucial service for adults with Down syndrome and will become more important from a public health perspective as large cohorts of adults with Down syndrome age into midadulthood.

Despite the importance of Medicaid for adults with Down syndrome, little is known about their patterns in enrollment, service use, and cost to the Medicaid program. Studies examined service use for adults with Down syndrome in 1 state,^11^ a small number of states,^12^ or among adolescents with Down syndrome,^13,14^ but characterizing health service use in a nationwide sample of adults in the US with Down syndrome has not been done, to our knowledge. For individuals with Down syndrome, understanding health and health service patterns are important for identifying subgroups that are not receiving optimal care, be it rural populations far from clinics^15^ or younger adults transitioning to adult health care services.^16^ Reckoning with racial disparities in health care for adults with Down syndrome is desperately needed. Death records show that Black adults with Down syndrome die earlier than White adults with Down syndrome,^3^ yet little else is known about the health and well-being of adults in racial and ethnic minority groups with Down syndrome in the US. Many Down syndrome cohorts are predominantly White and come from families with higher socioeconomic status.^17,18^ Medicaid data represent the full spectrum of adults with Down syndrome and can be used to identify racial and ethnic disparities in care^19,20,21^ and to inform the design and delivery of health interventions.^22^

It is important to examine how Medicaid, a federal-state partnership, serves adults with Down syndrome at a national and state level to identify service system gaps and state policies worth replicating. Individuals with disabilities comprise the largest proportion of Medicaid spending by enrollment group, totaling more than $195 billion in 2019.^23^ For individuals with Down syndrome, the potential benefit of preventive and appropriate care can be a large cost saving for Medicaid. For example, a study in South Carolina found that adults with intellectual disability experienced more than 21 000 potentially avoidable visits to the emergency department and had more than $35 million in health care costs due to ambulatory care–sensitive conditions, which are often overlooked in primary care.^24^ Understanding the demographic characteristics and enrollment and health care use patterns for adults with Down syndrome will be useful for planning, administering, and adapting health interventions and practices that can improve health for those with Down syndrome and the effectiveness of the Medicaid program.

Our objective was to describe enrollment in, health care use in, and cost to Medicaid for all adults 18 years of age or older with Down syndrome between 2011 and 2019 using data from the Centers for Medicare & Medicaid Services. We compared enrollment and service use for adults with Down syndrome with adults with intellectual disability without Down syndrome and a random sample of Medicaid-enrolled adults without developmental disabilities to examine whether results were not unique to individuals with Down syndrome but were associated with intellectual disability and to place patterns within the context of all Medicaid enrollees. We also explored differences in service use by race and ethnicity among enrollees with Down syndrome.

## Methods

### Data Source

In this cohort study, the data are from the Down Syndrome Toward Optimal Trajectories and Health Equity using Medicaid Analytic eXtract project (DS-TO-THE-MAX). The DS-TO-THE-MAX is a longitudinal claims database of all adults 18 years of age or older enrolled in Medicaid at any point between January 1, 2011, and December 31, 2019, with any 1 or more inpatient claim or 2 or more outpatient claims for Down syndrome, autism, or intellectual disability (see eAppendix 1 in Supplement 1 for a list of *International Classification of Diseases, Ninth Revision* and *International Statistical Classification of Diseases and Related Health Problems, Tenth Revision* codes) and a random sample of enrollees without developmental disability. Case definitions were based on previous literature.^11,25^ For these analyses, case definitions used Down syndrome as the primary case definition (ie, no one in the intellectual disability group had Down syndrome). Data include demographic files; inpatient, other service, and long-term care claims and encounters; and pharmacy prescription claims. Data were purchased from the Centers for Medicare & Medicaid Services after application and approved data use agreement. More information about the data acquisition process can be found online.^26^ This project was deemed exempt and participant consent was waived by the Boston University Medical Campus institutional review board as the deidentified data were considered nonhuman participants research. We followed the Strengthening the Reporting of Observational Studies in Epidemiology (STROBE) reporting guideline (eAppendix 3 in Supplement 1).

### Aligning Data Collection Systems

The DS-TO-THE-MAX spans 2 data collection systems: Medicaid Analytic eXtract (MAX; 2011-2015) and the Transformed Medicaid Statistical Information System Analytic Files (TAF; 2014-2019). There was a transitionary period in 2014 and 2015 with some states using MAX, whereas other states had already transitioned to TAF. A unique beneficiary identifier linked the 2 data systems. Many demographic variables had the same format and were checked for consistency. Other variables were reparameterized to align in both systems. Further information is provided in eAppendix 2 in Supplement 1.

### Demographic Data

We assessed demographic characteristics from the person file and demographic enrollment file. Age was determined by date of birth. Race and ethnicity were self-reported, and collection varied by state and year. If race or ethnicity were reported in any year, we considered that to be the individual’s race or ethnicity, even if other years had missing race or ethnicity information. Race was categorized as Asian, Black, Native American, Pacific Islander, multiple races (≥2 races reported), and White. We dichotomized ethnicity to Hispanic and non-Hispanic. If race was missing in all years, we used multiple imputation to probabilistically account for missingness. We used the American Community Survey 5-year summary data file^27^ to calculate the percentage of each race category at the zip code level. These proportions and demographic data were used for imputing the missing race and ethnicity. We did 30 imputations of a model that included age, sex, disability eligibility, dual enrollment, and zip code–level race and ethnicity distribution (preimputation data are presented in eTable 1 in Supplement 1).

### Enrollment Data

Data were linked across years by beneficiary identifier. We examined the number of individuals enrolled and person-time. An individual’s person-time would count toward the Down syndrome or intellectual disability group in any year they were enrolled if they had a Down syndrome or intellectual disability claim in any year (eg, if the first Down syndrome claim was in 2012 and they were enrolled in 2011, their 2011 person-time would be in the Down syndrome cohort). We used this approach because Down syndrome and intellectual disability are lifelong conditions, and a lack of claim likely indicates limited health care use in the year or clinician coding practice. If an individual turned 18 years of age after 2011, their data for years that they were younger than 18 years would be excluded. We calculated enrollment in 2 ways: ever enrolled in a year and months enrolled in the given year. We identified deaths while enrolled, loss to follow-up, and continuous enrollment.

Many adults with Down syndrome are eligible for Medicare regardless of age as Qualified Medicare Beneficiaries. We examined ever dual enrollment and person-time dual enrollment through yearly indicators of dual enrollment. Furthermore, we examined comprehensive managed care organization (MCO) enrollment and Medicaid eligibility source (disability or income) to characterize how and why individuals received care.

### Claims and Service Use

In our data, a claim or encounter represents documentation of a billed health care service. To evaluate interactions with the health care system, we examined mean and median number of claims per person-year. Claims were from the inpatient, other services, and long-term care files. In the MAX data, claims were summed across all files as part of the provided data sets, whereas in TAF, we calculated by counting the number of claims in each file for an individual.

We also evaluated hospitalizations because they are a commonly used prevention quality indicator.^28^ We created a count of hospitalizations based on unique claims with nonoverlapping admission dates and divided by person-years to calculate hospitalization rates. We used a similar approach to examine dates in long-term care facilities; for each unique nonoverlapping stay, we counted between admittance date and discharge date, then summed the total. For claims and service use variables, we age adjusted by age category so that the age distribution in the non–Down syndrome groups matched that of the Down syndrome group.

### Cost

We determined the amount Medicaid paid per individual per person-year. The Medicaid paid amount is the amount of money that the Medicaid system paid the clinician and does not reflect the original billed amount or out-of-pocket expenses. We included capitated payments to account for services provided by MCOs.^29^ The total cost was an existing variable in the MAX data and was derived from each file in the TAF data. We adjusted costs for inflation using the Consumer Price Index.^30^ We also calculated age-adjusted and inflation-adjusted costs for each group by file type (inpatient, outpatient, long-term care, and prescription drug).

### Statistical Analysis

Statistical analyses were conducted from June 2022 to February 2023. We calculated descriptive data for demographic characteristics and aggregate data by diagnostic group. We graphed the percentage of beneficiaries at each age by study year and diagnostic group. We calculated age-standardized death rates, claims, Medicaid paid costs per year, inpatient hospitalizations, and number of days in long-term care, with the Down syndrome group as the referent age distribution. Because of our large sample size that represents a near-full population sample, clinically insignificant results would be statistically significant; therefore, we do not present statistical tests because any minor difference would be statistically significant.^31^ We calculated age-adjusted claims and costs rates for the Down syndrome cohort by race and ethnicity and compared them with the White and non-Hispanic groups as referents. We conducted sensitivity analysis by accounting for reported poor data quality in some states in some years by comparing mean claims and costs for all states with only those with good data quality as reported by DQ Atlas.^32^

## Results

Our sample of Medicaid-enrolled adults from 2011 to 2019 included 123 024 unique individuals with Down syndrome (820 273 person-years of coverage; mean [SD] age, 35 [14.7] years; median age, 33 years [IQR, 21-48 years]; 51.6% men and 48.4% women; 14.1% Black individuals; 16.7% Hispanic individuals; and 74.6% White individuals), 1 182 246 unique individuals with intellectual disability (mean [SD] age, 37.1 [16.8] years; median age, 33 years [IQR, 22-50 years]; 56.5% men and 43.5% women; 22.0% Black individuals; 11.7% Hispanic individuals; and 69.5% White individuals), and 3 176 371 unique individuals with no diagnoses of developmental disabilities (mean [SD] age, 38 [18.6] years; median age, 33 years [IQR, 21-52 years]; 43.8% men and 56.2% women; 23.7% Black individuals; 20.7% Hispanic individuals; and 61.3% White individuals) (Table 1). There were 6602 individuals with Down syndrome and a dual diagnosis of autism (5.4% of all persons with Down syndrome) and 176 726 individuals with intellectual disability and a dual diagnosis of autism (14.9% of all individuals with intellectual disability). Age distributions over time are presented in Table 2.

**Table 1.** Demographic Characteristics for Adult Medicaid Enrollees With Down Syndrome or Intellectual Disability and a Sample of Medicaid Enrollees Without Developmental Disability, 2011-2019

Characteristic	Adult enrollees, No. (%)		
	Down syndrome (n = 123 024)	Intellectual disability (n = 1 182 246)	No developmental disability (n = 3 176 371)
Sex			
Female	59 600 (48.4)	514 653 (43.5)	1 785 963 (56.2)
Male	63 424 (51.6)	667 587 (56.5)	1 389 960 (43.8)
Race^a^			
Asian	4029 (3.4)	28 963 (2.6)	187 571 (6.1)
Black	16 826 (14.1)	249 635 (22.0)	723 538 (23.7)
Multiple races	7085 (5.9)	49 795 (4.4)	164 183 (5.4)
Native American	1034 (0.9)	9473 (0.8)	39 711 (1.3)
Pacific Islander	1258 (1.1)	8964 (0.8)	67 045 (2.2)
White	88 965 (74.6)	789 184 (69.5)	1 873 446 (61.3)
Missing	3829	46 232	120 877
Ethnicity			
Hispanic or Latino	19 876 (16.7)	133 207 (11.7)	631 393 (20.7)
Non-Hispanic or non-Latino	99 319 (83.3)	1 002 807 (88.3)	2 424 101 (79.3)
Unknown or missing	3829	46 232	120 877
Region			
Midwest	28 085 (22.8)	297 373 (25.2)	625 529 (19.7)
Northeast	27 436 (22.3)	295 400 (25.0)	638 376 (20.1)
South	38 932 (31.6)	317 318 (31.4)	1 022 386 (32.2)
West	26 782 (21.8)	212 358 (18.0)	802 505 (25.3)
US territory or other	656 (0.5)	5789 (0.5)	46 050 (1.4)
Eligibility type (ever)			
Disability	99 543 (80.9)	941 641 (79.7)	765 925 (24.1)
Income	57 939 (47.1)	624 931 (52.9)	1 695 248 (53.4)
Continuously enrolled	77 861 (63.3)	763 029 (64.5)	956 748 (30.0)
Loss to follow-up	13 214 (10.7)	153 520 (13.0)	1 384 630 (43.6)
Total person-years	820 273	8 051 162	12 384 921
Median (IQR)	8.0 (5.0-9.0)	8.0 (5.0-9.0)	3.0 (2.0-6.0)
Mean (SD)	6.6 (2.6)	6.6 (2.6)	3.3 (2.6)
Categorized age, person-years			
18-25	183 598	1 712 598	3 070 001
26-34	173 166	1 666 449	2 499 599
35-44	149 398	1 319 183	1 933 068
45-54	171 413	1 363 798	1 683 095
55-64	116 992	1 185 660	1 560 816
65-89^b^	26 504	807 913	1 726 305
No. of deaths (per 10 000 person-years)	20 094 (244.8)	114 861 (147.0)	149 622 (119.9)
Ever enrolled in an MCO	98 045 (79.7)	941 738 (79.7)	2 622 558 (82.6)
Person-years in an MCO	514 736 (62.8)	4 846 147 (60.2)	7 649 128 (61.8)
Median (IQR)	4.0 (1.0-7.2)	4.0 (1.0-7.0)	1.7 (0.4-3.8)
Mean (SD)	4.2 (3.3)	4.1 (3.2)	2.4 (2.4)
Ever dually enrolled in Medicare	73 162 (59.5)	510 559 (43.2)	706 630 (22.2)
Person-years dual enrolled	471 592 (57.5)	4 337 694 (53.8)	2 911 670 (22.2)
Median (IQR)	4.0 (0.0-8.3)	2.3 (0.0-8.5)	0.0 (0.0-0.0)
Mean (SD)	3.8 (3.8)	3.9 (3.9)	0.9 (2.2)

Abbreviation: MCO, managed care organization.

^a^ Multiple imputation used for missing race and ethnicity (25% missing race, 12% missing ethnicity; see eTable 1 in Supplement 1 for preimputation distribution). Data are still missing if they did not have zip code information.

^b^ Age older than 89 years listed as 89 for deidentification.

**Table 2.** Age Distributions by Year and Change Over 9 Years in Medicaid Enrollees With DS or ID, or Without DD, 2011-2019

Year	% of Enrollees by age group																	
	18-25 y			26-34 y			35-44 y			45-54 y			55-64 y			≥65 y		
	DS	ID	No DD	DS	ID	No DD	DS	ID	No DD	DS	ID	No DD	DS	ID	No DD	DS	ID	No DD
2011	22	22	25	18	19	19	19	17	15	25	19	14	13	14	11	3	9	16
2012	22	23	25	19	19	19	18	16	15	24	19	14	14	14	11	3	9	16
2013	23	22	25	19	20	19	18	16	15	23	18	13	14	14	11	3	9	16
2014	23	22	24	20	20	20	18	16	16	22	18	14	14	15	13	3	10	14
2015	23	22	24	21	20	20	18	16	16	21	17	14	14	15	13	3	10	13
2016	23	22	24	22	21	21	18	16	16	20	16	14	15	15	13	3	10	13
2017	23	21	24	22	22	21	18	16	16	19	16	13	15	15	13	3	11	13
2018	22	20	25	23	22	20	18	17	16	18	15	13	15	15	13	3	11	13
2019	21	18	26	24	23	20	19	17	15	17	15	12	15	15	13	4	11	14
% Change, 2011-2019	−4.5	−18.2	4.0	33.3	21.1	5.3	0	0	0	−32.0	−21.1	−14.3	15.4	7.1	18.2	33.3	22.2	−12.5

Abbreviations: DD, developmental disability; DS, Down syndrome; ID, intellectual disability.

Over the 9 years studied, Medicaid insured 820 273 person-years for adults with Down syndrome, with a median enrollment length of 8.0 years (IQR, 5.0-9.0 years) and a mean (SD) enrollment length of 6.6 (2.6) years (Table 1). In total, 20 094 adults with Down syndrome died during the study period (244.8 deaths per 10 000 person-years). After age standardizing to the Down syndrome sample, the death rates of the intellectual disability group (131.2 deaths per 10 000 person-years) and the no developmental disability group (76.7 deaths per 10 000 person-years) were all considerably lower than the death rate of the Down syndrome group. A total of 59.5% of adults with Down syndrome and 43.2% of adults with intellectual disability were ever dually enrolled in Medicare, with more than 88.0% of those 45 years or older at study entry ever being dually enrolled. Regarding MCO coverage, 79.7% of enrollees with Down syndrome or intellectual disability were ever enrolled in an MCO, while 82.6% of enrollees in the random sample were ever enrolled. There was an increasing trend over time (2011: 53.2% and 68.8% MCO use in the Down syndrome and no developmental disability cohorts, respectively; 2019: 76.0% and 81.1% MCO use in the Down syndrome and no developmental disability cohorts, respectively).

Medicaid-enrolled adults with Down syndrome had more than 3 times the claims of the age-adjusted, no developmental disability group (median, $26 278 per person-year [IQR, $11 145-$55 928 per person-year] vs $6173 per person-year [IQR, $5811-$8757 per person-year]) (Table 3). Costs were higher for the Down syndrome group relative to the no developmental disability group but lower than for the intellectual disability group. When disaggregated, the Down syndrome group had higher long-term care costs than the intellectual disability group. In a sensitivity analysis restricted to states with high data quality in TAF, we found minimal changes in claims and costs compared with including all states (eTable 2 in Supplement 1). After age adjustment, the Down syndrome and intellectual disability groups had similar hospitalization rates compared with the no developmental disability group. There were few qualitative differences when comparing outcomes among MAX (2011-2013), MAX and TAF (2014-2015), and TAF (2016-2019) (eTable 3 in Supplement 1).

**Table 3.** Medicaid Service Use and Cost, 2011-2019

Characteristic	Down syndrome (820 273 person-years)		Intellectual disability (8 051 162 person-years)		No developmental disability (12 384 921 person-years)	
	Mean (SD)	Median (IQR)	Mean (SD)	Median (IQR)	Mean (SD)	Median (IQR)
Claims per person-year						
Unadjusted	205 (201)	131 (65-277)	223 (216)	146 (72-301)	52 (85)	30 (11-62.)
Age adjusted^a^	[Reference]	[Reference]	231 (253)	238 (108-284)	64 (77)	53 (48-78)
Medicaid paid costs per person-year, $^b^						
Unadjusted	42 515 (47 397)	26 278 (11 145-55 928)	52 291 (64 472)	31 868 (11 800-68 471)	6636 (16 178)	3703 (868-58 390)
Age adjusted	[Reference]	[Reference]	53 893 (70 779)	50 466 (35 007-57 706)	7545 (18 764)	6173 (5811-8757)
Disaggregated costs, $^c^						
Inpatient	2143 (11 067)	251 (3-810)	878 (8767)	850 (831-1131)	581 (6561)	430 (334-1030)
Outpatient	27 566 (28 809)	18 305 (7160-46 607)	40 123 (12 829)	41 196 (31 091-45 076)	5900 (3384)	5237 (4874-7692)
Long-term care	42 113 (60 759)	23 238 (2655-56 140)	11 503 (35 895)	6682 (4611-20 383)	582 (15 345)	223 (162-1311)
Prescription drugs	841 (5160)	50 (5-269)	1392 (3847)	1740 (830-2147)	488 (4102)	348 (116-769)
Inpatient hospitalizations per 1000 person-years, median (IQR)						
Unadjusted	20.4 (55.5)	0	25.9 (78.9)	0	16.1 (60.4)	0
Adjusted	[Reference]	[Reference]	22.7 (105.2)	0	16.9 (79.4)	0
No. of days in long-term care per person-year						
Unadjusted	33 (101)	0	43 (122)	0	6 (44)	0
Adjusted	[Reference]	[Reference]	40 (101)	0	4 (16)	0

^a^ Adjusted estimates are age standardized to the Down syndrome cohort.

^b^ Dollar estimates adjusted for inflation.

^c^ Disaggregated costs adjusted for inflation and age standardized for the intellectual disability and no developmental disability groups. Costs differ compared with total costs because disaggregate costs are mean costs within a category and do not account for correlation between cost categories.

When examining costs and claims per person-year in the Down syndrome cohort by race and ethnicity, White enrollees had more costs than enrollees from other racial and ethnic groups, with the largest difference in the Asian and Pacific Islander enrollee groups (Table 4). White enrollees with Down syndrome had more claims after age adjustment than enrollees from all other racial and ethnic groups. Pacific Islander enrollees with Down syndrome had the lowest claim and cost rates.

**Table 4.** Claims and Costs per Person-Year for Adults With Down Syndrome Enrolled in Medicaid by Race and Ethnicity, 2011-2019^a^

Characteristic	Claims		Costs	
	Per person-year	Ratio	Per person-year, $	Ratio
Race				
Asian	172.9	0.78	40 538	0.87
Black	211.2	0.95	38 445	0.82
Mixed race	190.8	0.86	37 575	0.80
Native American	187.5	0.84	43 269	0.92
Pacific Islander	170.0	0.76	32 912	0.70
White	222.0	1 [Reference]	46 845	1 [Reference]
Ethnicity				
Hispanic	183.6	0.83	39 589	0.82
Non-Hispanic	222.1	1 [Reference]	48 182	1 [Reference]

^a^ Age standardized with White as reference group. Multiple imputation was used for missing race and ethnicity (25% missing race, 12% missing ethnicity).

## Discussion

Individuals with Down syndrome can now expect to live into their 60s and consequently use Medicaid to manage their high health care needs. To understand how to best serve and provide care for the adult population with Down syndrome, we have created a longitudinal cohort of 123 024 adults with Down syndrome in the Medicaid system from 2011 to 2019. This sample—to our knowledge, an order of magnitude larger and more racially representative than other samples—enables us to understand gaps in enrollment, health care use, and cost and to characterize racial and ethnic disparities.

Taking into account projections by de Graaf et al^2^ of the full adult population with Down syndrome in the US (approximately 125 000), Medicaid is the majority insurance provider for all adults with Down syndrome. A proportion of these adults are likely Medicare enrolled without dual Medicaid eligibility because they are older than 65 years or have a parent who meets Medicare eligibility.^33^ Only a small proportion of adults with Down syndrome have full-time employment that provides employer-subsidized health insurance. In 1 online survey of 511 people with Down syndrome and/or their caregivers in the US, only 3% reported working more than 30 hours per week.^34^ Despite the clear association of employment with mental and physical health,^35^ there are negative incentives for employment for adults with disabilities because increased wages may reduce Social Security benefits.^36^ We saw an increasing enrollment in Medicaid for adults with Down syndrome over time, highlighting the continued and growing importance of Medicaid in facilitating access to care over individuals’ lifetime.

Adults with Down syndrome are more consistently enrolled in Medicaid with less churn (ie, pattern of disenrollment and reenrollment) compared with the population without developmental disabilities, possibly due to automatic Medicaid enrollment mechanisms for individuals meeting financial and medical criteria used by many states. With such consistent insurance coverage, adults with developmental disabilities are potentially less likely to experience the care disruptions and poor outcomes associated with health insurance churn.^37^ Continuity within the Medicaid program highlights how claims data are a valuable resource for Down syndrome research because the risk of bias due to missing data and loss to follow-up are minimal. These data are pivotal for evaluating Medicaid policy and allocation of services in disabled populations. Based on the frequency of dually eligible enrollment in Medicare, Medicare is also an important insurer of adults with Down syndrome, and data are needed to supplement Medicaid claims when asking questions about health and total service use among adults with Down syndrome.

In our data, costs paid by Medicaid for adults with Down syndrome and claims to the Medicaid system were lower compared with those for adults with intellectual disability without Down syndrome and were greater compared with our random no developmental disability group. The mean cost and number of claims were in line with previous work estimating Medicaid costs in the Chicago area from 2010 to 2011^38^ but were lower than previous work in Wisconsin Medicaid from 2011 to 2019.^11^ Costs and claims likely vary between states due to policy differences, such as Medicaid expansion,^39^ data quality,^32^ and percentage of services covered by MCOs.^38^ Based on consistent enrollment and clear eligibility, we hypothesize that these issues and policies impact data for adults with Down syndrome less than peers. Nevertheless, adults with Down syndrome use a substantial amount of Medicaid-covered services. Compared with people without disabilities, outpatient long-term care costs are associated with the higher cost for people with Down syndrome or intellectual disability. With the high prevalence of chronic conditions among adults with Down syndrome,^4^ a focus on preventive care may help deter avoidable acute care.^40^

### Limitations

This study has some limitations. Some data are missing by state and year, and the Centers for Medicare & Medicaid services report data quality issues for some data sources for some states in some years.^32^ Because of the consistent enrollment in Medicaid of adults with Down syndrome in our data, we were able to use all 9 years of data to impute demographic data. We examined our data by year to evaluate any patterns in missing claims and found no difference by year. There are reported inconsistencies in costs, especially surrounding MCOs; we present these data with sensitivity analyses that illustrate robustness. Ultimately, our results may underestimate the true amounts of claims, costs, and hospitalizations, but with the given data infrastructure, these estimates are a large advancement. The way data were collected and coded differed between MAX and TAF, and while we aligned the 2 data sets, some variable coding did not perfectly align. We compared MAX and TAF in sensitivity analysis and found little difference in enrollment and median costs and claims.

## Conclusions

This cohort study found that the Medicaid program plays a key role in insuring adults with Down syndrome and facilitating access to health services for a community with high health care needs and costs. Given the consistent and near-universal enrollment in Medicaid of individuals with Down syndrome, Medicaid data are a useful tool for understanding the health and well-being of individuals with Down syndrome.

## Notes

Supplement 1. **eAppendix 1.** ICD-9 and ICD-10 Codes Used to Identify Down Syndrome and Intellectual Disability **eAppendix 2.** Additional Data Processing Detail **eAppendix 3.** STROBE Statement—Checklist of Items That Should be Included in Reports of Cohort Studies **eTable 1.** Impact of Multiple Imputation on Racial and Ethnic Distribution, by Disability Cohort **eTable 2.** Sensitivity Analysis Assessing Restricting to States With “Good” Data Quality as Reported by DQ Atlas **eTable 3.** Main Outcomes by Year to Assess Changes Across Data Collection Practices

Supplement 2.
Data Sharing Statement
