# Supplementary material for: Medicaid Enrollment and Service Use Among Adults With Down Syndrome
Source: JAMA Health Forum. 2023 Aug 11;4(8):e232320. doi: 10.1001/jamahealthforum.2023.2320 (PMC10422190; doi:10.1001/jamahealthforum.2023.2320)
Supplement: Supplement 1. — eAppendix 1. ICD-9 and ICD-10 Codes Used to Identify Down Syndrome and Intellectual Disability eAppendix 2. Additional Data Processing Detail eAppendix 3. STROBE Statement—Checklist of Items That Should be Included in Reports of Cohort Studies eTable 1. Impact of Multiple Imputation on Racial and Ethnic Distribution, by Disability Cohort eTable 2. Sensitivity Analysis Assessing Restricting to States With “Good” Data Quality as Reported by DQ Atlas eTable 3. Main Outcomes by Year to Assess Changes Across Data Collection Practices [file jamahealthforum-e232320-s001.pdf]

Supplementary Online Content

Rubenstein E, Michals A, Wang N, et al. Medicaid enrollment and service use among adults with Down syndrome. *JAMA Health Forum.* 2023;4(8):e232320. doi:10.1001/jamahealthforum.2023.2320

eAppendix 1. ICD-9 and ICD-10 Codes Used to Identify Down Syndrome and Intellectual Disability
eAppendix 2. Additional Data Processing Detail
eAppendix 3. STROBE Statement—Checklist of Items That Should be Included in Reports of Cohort Studies
eTable 1. Impact of Multiple Imputation on Racial and Ethnic Distribution, by Disability Cohort
eTable 2. Sensitivity Analysis Assessing Restricting to States With "Good" Data Quality as Reported by DQ Atlas
eTable 3. Main Outcomes by Year to Assess Changes Across Data Collection Practices

This supplementary material has been provided by the authors to give readers additional information about their work.

© 2023 Rubenstein E et al. *JAMA Health Forum.*

eAppendix 1. ICD-9 and ICD-10 Codes Used to Identify Down Syndrome and Intellectual Disability

	ICD9	ICD10
Down syndrome	758	Q90
		Q90.0
		Q90.1
		Q90.2
		Q90.9
Intellectual disability	F70	317
	F71	318
	F72	318
	F73	318.1
	F78	318.2
	F79	319

© 2023 Rubenstein E et al. *JAMA Health Forum.*

eAppendix 2. Additional Data Processing Detail

Data were sent to study team in files by year, where data cleaning relied entirely on source type: MAX or TAF. The two systems were very different in how they were structured, in both size and number of files per source. MAX data structure was smaller (in size and number) than TAF, where data were one file per inpatient, outpatient, long-term care, and medication source. TAF had up to three files per source, requiring data processing to cover multiple files in order to accurately combine measures.

We kept the yearly data structure for demographic cleaning in order to have a full record of yearly data for each individual in our cohort. Using claim lines with a non-missing bene_id, we assigned individuals to the DS, AS, and ID cohorts by flagging claims with a predetermined list of ICD codes. We retained individuals in the yearly data by flagging those having 1 or more inpatient claim or 2 or more claims from other sources. The next step was to combine with the random sample group. For demographics, we had CMS-regulated exclusion criteria for age of 18 to 89 years old. In order to QC this, we first cut the PS file for MAX and Demographic file for TAF by these age criteria. Then, we merged this sample to the sub-cohort we created by bene_id. All individuals were controlled for age before being brought into the dataset so no restricted ages could be included.

The next step in creating yearly datasets was to combine the MAX and TAF data that was split for years 2014 and 2015, as CMS transitioned to the new data structure during this time. Therefore, we had two files for each year. The biggest QC priority when linking was to consider participants moving within the year. If this happened, there were multiple rows per individual to account for the data recorded in different states. In almost every case, the person's data did not stop being reported at their prior state of residence while also starting and continuing in their current state of residence at the same time (i.e., a person would have up to 2 states claiming they were enrolled at once). For this reason, processing insurance/MCO categorizations, eligibility types, and dual enrollment required careful attention and cross referencing. We used non-missing data for the most important demographic information, i.e. DS, AS, ID indicators and counts, race, gender, and age. For all other variables (enrollment months, eligibility type, medically needy, MCO ever, etc.), we used the most recent data available. Variables that were counts, like enrollment months, were summed between the data.

Demographics were created by cross referencing the CMS provided data dictionaries for MAX and TAF to ensure the most similar variables were being used to calculate the same derived variables. The largest portion of QC at this stage was comparing the two sources for the creation of derived variables. All derived variables were structured/created the same across data sources for use in analysis. A main difference between the data structures was that TAF reported more monthly measurements, MAX reported more annual measurements, and depending on the variable, one system would require a measurement to be calculated while the other contained it in the raw data. For example, age needed to be calculated in MAX but is included as a demographic variable in TAF. We needed to ensure how CMS calculated variables that MAX did not contain, or vice versa, to ensure accurate measurement of demographics between the two.

Our demographics were based on the first year of recorded claims for that individual. However, due to the nature of the conditions we focused on, we categorized our cohort groups as ever having them indicated in their data over the nine years (if someone was ever flagged for DS codes, they were categorized as such for the study period). During demographic data cleaning, we discovered that some individuals had multiple races and ethnicities reported throughout the years. We cleaned this by flagging any individual with more than one race listed and categorizing them as 'Multiple Races', saving the listed races as variables for use in later sensitivity analyses. Additionally, if someone's first year of data listed race as 'Unknown/Missing' but listed a constant known race for later years, we used the race indicated in later years. For ethnicity, we considered with any indication of Hispanic ethnicity to have Hispanic ethnicity regardless of ever having reported white ethnicity. However, if they did have both, we created a flag for future sensitivity analyses. There were many individuals whose claims were missing sex categorizations. To clean this if a person's first year of data was missing this variable but later years were not, we used the first non-missing data available.

For analysis of claims, costs, and visits, the measures were calculated by CMS and contained in the MAX PS file. For TAF, these measures needed to be hand calculated across all data sources. In order to QC total claims, costs, inpatient hospitalizations, and long-term care days in TAF and ensure the two data types were processed the same way, we replicated the entire program used to calculate TAF to hand calculate the same variables in MAX, since

© 2023 Rubenstein E et al. *JAMA Health Forum.*

they were supplied by CMS. We aggregated the applicable data from all file types to sum these measures by person, then compared the total mean and other descriptive to that of the descriptive in the PS file. Once we confirmed this method gave near identical results as the data given by CMS, we processed all TAF data this way. In order to remove outliers, we used the formula [mean+2(standard deviation)] for the upper limit. The maximum number of outliers removed was ~200 for any given year.

In order to accurate calculate total cost across data types (IP, OP, LTC, and Rx), we wanted a way to QC our programming to replicate what the summary file, PS, for MAX reports. For total costs, MAX provided yearly totals (i.e. aggregate costs for entire year) in the PS dataset. TAF did not supply any aggregate data, meaning we needed to sum claim counts, cost, IP stays, and LTC days by hand. To QC this for TAF, after building the program to sum these variables in each TAF dataset, we processed the separate MAX files using the same program to know how close our method was to what CMS used to report the yearly totals. We compared the distributions of the PS dataset for claim count, cost, IP and LTC days to the distributions calculated from the TAF program and found they were only different by a maximum of 1.6 for claim count, $323.7 for cost and 0.02 for IP stays. The LTC day count was the same. Since our TAF program compiled so closely to the MAX summary distributions, our processing plan was the best method.

MAX 2011 Table 3 Distributions: Using PS File

Label	N	Mean	Std Dev	Minimum	Maximum
Total Claims 2011	2503444	124.1945192	191.2165387	0	4769.00
Total Cost 2011	2503444	24272.99	59668.42	0	6666714.00
Total IP Stays 2011	2503444	0.1803104	0.9228653	0	153.0000000
Total LTC Days 2011	182414	268.3598189	162.1505457	-360.0000000	4614.00

MAX 2011 Table 3 Distributions: manually created using IP, LTC, OP, Rx files (QC/TAF Method)

Total Claims 11 QC	2471108	125.8195696	191.9315819	0	4769.00
Total Cost 11 QC	2471107	24596.70	59992.81	0	6666714.00
Total IP Stays 11 QC	2470845	0.1608814	0.7327922	0	133.0000000
Total LTC Days 11 QC	182414	268.3598189	162.1505457	-360.0000000	4614.00

To accurate calculate total cost across data types (IP, OP, LTC, and Rx), we wanted a way to QC our programming to replicate what the summary file, PS, for MAX reports. For total costs, MAX provided yearly totals (i.e., aggregate costs for entire year) in the PS dataset. TAF did not supply any aggregate data, meaning we needed to sum claim counts, cost, IP stays, and LTC days by hand. To QC this for TAF, after building the program to sum these variables in each TAF dataset, we processed the separate MAX files using the same program to know how close our method was to what CMS used to report the yearly totals. We compared the distributions of the PS dataset for claim count, cost, IP and LTC days to the distributions calculated from the TAF program and found they were only different by a maximum of 1.6 for claim count, $323.7 for cost and 0.02 for IP stays. The LTC day count was the same. Since our TAF program compiled so closely to the MAX summary distributions, our processing plan was the best method.

We then adjusted each year's total cost for inflation by using historical, national CPI data for the years 2011-2019. Total costs for each year were adjusted by that year's CPI using [cost*(1+ inflation rate)]. We also standardized by age using DS cohort's age category frequencies for each year of data. We calculated the frequencies of age categories for the DS cohort by year, then multiplied the total measures (cost, claims, IP stays, long-term care days) by the percent in the corresponding age strata for all other cohort groups.

© 2023 Rubenstein E et al. *JAMA Health Forum.*

eAppendix 3. STROBE Statement—Checklist of Items That Should be Included in Reports of Cohort Studies

	Item No	Recommendation	Page No
**Title and abstract**	1	(*a*) Indicate the study's design with a commonly used term in the title or the abstract	1
		(*b*) Provide in the abstract an informative and balanced summary of what was done and what was found	3
**Introduction**			
Background/rationale	2	Explain the scientific background and rationale for the investigation being reported	5-6
Objectives	3	State specific objectives, including any prespecified hypotheses	7
**Methods**			
Study design	4	Present key elements of study design early in the paper	8
Setting	5	Describe the setting, locations, and relevant dates, including periods of recruitment, exposure, follow-up, and data collection	8
Participants	6	(*a*) Give the eligibility criteria, and the sources and methods of selection of participants. Describe methods of follow-up	8
		(*b*) For matched studies, give matching criteria and number of exposed and unexposed	
Variables	7	Clearly define all outcomes, exposures, predictors, potential confounders, and effect modifiers. Give diagnostic criteria, if applicable	9-11
Data sources/ measurement	8*	For each variable of interest, give sources of data and details of methods of assessment (measurement). Describe comparability of assessment methods if there is more than one group	9
Bias	9	Describe any efforts to address potential sources of bias	11
Study size	10	Explain how the study size was arrived at	n/a
Quantitative variables	11	Explain how quantitative variables were handled in the analyses. If applicable, describe which groupings were chosen and why	11
Statistical methods	12	(*a*) Describe all statistical methods, including those used to control for confounding	11
		(*b*) Describe any methods used to examine subgroups and interactions	11
		(*c*) Explain how missing data were addressed	9
		(*d*) If applicable, explain how loss to follow-up was addressed	
		(*e*) Describe any sensitivity analyses	11
**Results**			
Participants	13*	(a) Report numbers of individuals at each stage of study—eg numbers potentially eligible, examined for eligibility, confirmed eligible, included in the study, completing follow-up, and analysed	12-13
		(b) Give reasons for non-participation at each stage	n/a
		(c) Consider use of a flow diagram	n/a
Descriptive data	14*	(a) Give characteristics of study participants (eg demographic, clinical, social) and information on exposures and potential confounders	13, 22
		(b) Indicate number of participants with missing data for each variable of interest	22
		(c) Summarise follow-up time (eg, average and total amount)	22
Outcome data	15*	Report numbers of outcome events or summary measures over time	23

© 2023 Rubenstein E et al. *JAMA Health Forum.*

Main results	16	(*a*) Give unadjusted estimates and, if applicable, confounder-adjusted estimates and their precision (eg, 95% confidence interval). Make clear which confounders were adjusted for and why they were included<br>(*b*) Report category boundaries when continuous variables were categorized<br>(*c*) If relevant, consider translating estimates of relative risk into absolute risk for a meaningful time period	23
Other analyses	17	Report other analyses done—eg analyses of subgroups and interactions, and sensitivity analyses	14
**Discussion**			
Key results	18	Summarise key results with reference to study objectives	14
Limitations	19	Discuss limitations of the study, taking into account sources of potential bias or imprecision. Discuss both direction and magnitude of any potential bias	15
Interpretation	20	Give a cautious overall interpretation of results considering objectives, limitations, multiplicity of analyses, results from similar studies, and other relevant evidence	15-17
Generalisability	21	Discuss the generalisability (external validity) of the study results	15
**Other information**			
Funding	22	Give the source of funding and the role of the funders for the present study and, if applicable, for the original study on which the present article is based	18

*Give information separately for exposed and unexposed groups.

Note: An Explanation and Elaboration article discusses each checklist item and gives methodological background and published examples of transparent reporting. The STROBE checklist is best used in conjunction with this article (freely available on the Web sites of PLoS Medicine at http://www.plosmedicine.org/, Annals of Internal Medicine at http://www.annals.org/, and Epidemiology at http://www.epidem.com/). Information on the STROBE Initiative is available at http://www.strobe-statement.org.

© 2023 Rubenstein E et al. *JAMA Health Forum.*

eTable 1. Impact of Multiple Imputation on Racial and Ethnic Distribution, by Disability Cohort

		Non-imputed %			Imputed %, (Δ in %)		
		Down syndrome N=123024	Intellectual disability N=1182246	Random sample N=3176371	Down syndrome	Intellectual disability	Random sample
**Race**	Asian	2.6	1.9	5.4	3.4 (0.8)	2.6 (0.7)	6.1 (0.7)
	Black	13.9	23.1	26.1	14.1 (0.2)	22.0 (-1.1)	23.7 (-2.4)
	Native American	1.0	1.0	1.7	5.9 (4.9)	4.4 (3.4)	5.4 (3.7)
	Pacific Islander	0.4	0.4	1.0	0.9 (0.5)	0.8 (0.4)	1.3 (0.3)
	Multiple Races	1.4	1.7	1.3	1.1 (0.5)	0.8 (0.4)	2.2 (0.9)
	White	80.5	72.1	64.4	74.6 (-5.9)	69.5 (-2.6)	61.3 (-3.1)
	Unknown or missing (N)	28,920	198,854	951,201	3,829	46,232	120,877
**Ethnicity**	Hispanic/Latino	17.1	11.7	21.4	16.7 (0.4)	11.7 (0.0)	20.7 (-0.7)
	Non-Hispanic/Latino	82.3	88.3	78.5	83.3 (1.0)	88.3 (0.0)	79.3 (0.8)
	Unknown or missing (N)	14,883	133,400	426,578	3,829	46,232	120,877

© 2023 Rubenstein E et al. *JAMA Health Forum.*

eTable 2. Sensitivity Analysis Assessing Restricting to States With "Good" Data Quality as Reported by DQ Atlas

	Down syndrome			Intellectual disability			Non Developmental disability		
	Full	Restricted	% Difference	Full	Restricted	% Difference	Full	Restricted	% Difference
Claim count (per person year)									
2014	224.5	225.9	0.6	240.8	242	0.5	61.3	61.5	0.3
2015	218.9	220.3	0.6	232.9	235.8	1.2	55.7	55.2	0.9
2016	202.3	214.3	5.9	216.5	223	3.0	58.8	59.7	1.5
2017	208.8	206.9	0.9	222.1	222.5	0.2	61.9	66.4	7.3
2018	221.3	217.4	1.8	232.7	230.6	0.9	64.3	68.8	7.0
2019	231.0	214.5	7.1	241.8	231.9	4.1	65.5	66.8	2.0
Cost ($)									
2014	43545	45634	4.8	52812	53861	1.99	7292	7589	4.1
2015	45237	51129	13.0	53304	56213.6	5.46	6840	7305.9	6.8
2016	42328	47910	13.2	51134	55310	8.17	7167	7778.7	8.5
2017	45232	44860	0.8	54147	52734.8	2.61	7702	8104.1	5.2
2018	47858	51677	8.0	57627	59340.9	2.97	8266	8743.1	5.8
2019	49927	49997	0.1	60447	61093	1.07	8803	8620.2	2.1

Groups are not age standardized and costs are not adjusted for inflation

© 2023 Rubenstein E et al. *JAMA Health Forum.*

eTable 3. Main Outcomes by Year to Assess Changes Across Data Collection Practices

	Down Syndrome			Intellectual Disability			Random Sample		
	Mean PY	SD	Median PY	Mean PY	SD	Median PY	Mean PY	SD	Median PY
Claims									
2011	202	237	104	218	251	119	62	108	27
2012	212	245	114	230	256	130	66	113	30
2013	216	239	119	236	257	136	68	115	31
2014	222	254	118	241	264	134	61	105	29
2015	215	248	111	233	262	127	56	95	26
2016	202	229	114	217	243	126	59	89	34
2017	209	223	122	222	238	133	62	88	36
2018	221	231	133	233	242	142	64	93	36
2019	231	239	138	242	250	147	66	97	37
Medicaid Paid Costs									
2011	43038	54698	26368	54402	93527	30525	7905	20430	2231
2012	42714	52921	27732	53568	90552	30908	7920	20005	2337
2013	42052	47978	27596	51750	65828	31218	7955	19037	2374
2014	44165	56312	27090	53647	72159	30280	7411	17863	2662
2015	44601	61488	24211	53407	72955	27730	6849	17415	2597
2016	42861	49733	26891	51779	62957	30239	7257	19339	2965
2017	46196	51185	31511	55300	66228	34202	7867	20271	3271
2018	49026	54652	34410	59033	69088	37374	8468	22080	3530
2019	50831	56689	34870	61541	71510	39520	8963	24850	3703
Inpatient Hospitalizations									
2011	0.16	0.68	0	0.23	1.10	0	0.21	0.96	0
2012	0.17	1.16	0	0.23	1.19	0	0.21	1.08	0
2013	0.17	0.87	0	0.23	1.20	0	0.21	1.08	0
2014	0.17	1.20	0	0.22	1.42	0	0.18	1.35	0
2015	0.16	0.61	0	0.23	0.99	0	0.15	0.72	0
2016	0.17	0.57	0	0.23	0.88	0	0.15	0.64	0
2017	0.18	0.59	0	0.24	0.86	0	0.15	0.62	0
2018	0.18	0.59	0	0.24	0.88	0	0.14	0.61	0
2019	0.19	0.64	0	0.25	0.94	0	0.14	0.60	0
Days in Long-Term Care									
2011	23	86	0	31	104	0	3	28	0
2012	23	89	0	30	105	0	3	31	0
2013	23	87	0	30	102	0	3	29	0
2014	25	99	0	32	111	0	3	34	0
2015	24	103	0	31	118	0	3	36	0
2016	28	130	0	39	156	0	3	43	0
2017	27	122	0	38	148	0	3	42	0
2018	28	125	0	40	151	0	3	41	0
2019	24	117	0	37	144	0	3	40	0

White: MAX data
Light gray: MAX-TAF transition
Dark gray: TAF data

© 2023 Rubenstein E et al. *JAMA Health Forum.*
