# Supplementary material for: Medicaid Enrollment and Service Use Among Adults With Down Syndrome
Source: JAMA Health Forum. 2023 Aug 11;4(8):e232320. doi: 10.1001/jamahealthforum.2023.2320 (PMC10422190; doi:10.1001/jamahealthforum.2023.2320)
Supplement: Supplement 2. — Data Sharing Statement [file jamahealthforum-e232320-s002.pdf]

Data Sharing Statement

Rubenstein. Medicaid Enrollment and Service Use Among Adults With Down Syndrome. *JAMA Health Forum*. Published August 11, 2023. doi:10.1001/jamahealthforum.2023.2320

Data
Data available: No

Additional Information
Explanation for why data not available: Data are used under a Data Use Agreement with the Center for Medicare and Medicaid Systems which does not allow data sharing. We can share a data dictionary but cannot share invidiual patient data
